# Validation of the Orlando Protocol for endoscopic management of pancreatic fluid collections in the era of lumen‐apposing metal stents

**DOI:** 10.1111/den.14099

**Published:** 2021-09-06

**Authors:** Ji Young Bang, C. Mel Wilcox, Juan Pablo Arnoletti, Shajan Peter, John Christein, Udayakumar Navaneethan, Robert Hawes, Shyam Varadarajulu

**Affiliations:** ^1^ Digestive Health Institute Orlando Health Orlando FL USA; ^2^ Department of Specialized Surgery AdventHealth Orlando Orlando FL USA; ^3^ Division of Gastroenterology and Hepatology University of Alabama at Birmingham Birmingham AL USA; ^4^ Department of Hepatobiliary and Pancreatic Surgery Grandview Cancer Center Birmingham USA

**Keywords:** lumen‐apposing metal stents, pancreatic fluid collection, plastic stents, pseudocyst, walled‐off necrosis

## Abstract

**Objectives:**

Although lumen‐apposing metal stents (LAMS) are being increasingly used in lieu of plastic stents, the clinical approach to endoscopic management of pancreatic fluid collections (PFCs) is poorly standardized. We compared outcomes of approaches over two time intervals, initially using plastic stents and later integrating LAMS.

**Methods:**

This was a retrospective, observational, before–after study of prospectively collected data on consecutive patients with symptomatic PFCs managed over two time periods. In the initial period (January 2010–January 2015) endoscopic treatment was undertaken with plastic stents and in the later period (February 2015–August 2020) by integration of LAMS with selective use of plastic stents. The treatment strategy in both periods were tailored to size, extent, type of PFC and stepwise response to intervention. The main outcome was treatment success, defined as resolution of PFC and presenting symptoms at 6‐month follow‐up.

**Results:**

A total of 160 patients were treated with plastic stents and 227 patients were treated using an integrated LAMS approach. Treatment success was significantly higher for the integrated approach compared to using only plastic stents (95.6 vs. 89.4%; *P* = 0.018), which was confirmed to be predictive of treatment success on multivariable logistic regression analysis (odds ratio 2.7, 95% confidence interval 1.1–6.4; *P* = 0.028).

**Conclusions:**

A structured approach integrating LAMS with selective use of plastic stents improved treatment success in patients with PFCs compared to an approach using only plastic stents.

## Introduction

Endoscopic drainage of pancreatic fluid collections (PFCs) is safe and clinically effective. The procedure entails creation of a fistulous tract between the PFC and gastrointestinal lumen by placement of an endoprosthesis. Depending on the degree of necrosis and extent of collection, a more lesion‐specific approach that entails additional interventions, such as creation of multiple transluminal tracts, percutaneous catheter drainage and endoscopic or percutaneous necrosectomy, may be required in a subset of patients.^1^, ^2^, ^3^ Regardless of the type of intervention undertaken, the first step in endoscopic management is placement of a transluminal endoprosthesis to facilitate drainage. While this objective was achieved historically by placement of 7‐Fr or 10‐Fr double pigtail plastic stents, more recently, given three unique advantages, lumen‐apposing metal stents (LAMS) have been increasingly used in lieu of plastic stents.^4^ First, the large diameter (10–20 mm) facilitates better drainage. Second, by virtue of their ‘apposing’ property, LAMS minimizes the risk of perforation and peritoneal leakage. Third, as the delivery system is constructed over a dedicated single‐step platform, stent deployment is technically easier. Despite these positive attributes, it is important to be cognizant of other clinical implications associated with use of LAMS in order to achieve optimal outcomes. Unlike plastic stents that can be left indwelling, LAMS need to be removed within 4 weeks to minimize adverse events.^5^, ^6^ Also, as disconnected pancreatic duct syndrome (DPDS) is frequently observed in necrotizing pancreatitis, LAMS must be exchanged for plastic stents in a timely manner, before the cavity seals completely, to minimize risk of PFC recurrence.^7^

While significant progress has been made with respect to the development of novel endoprostheses, treatment strategies in PFC management remain poorly standardized with no consensus on choice of procedural techniques, criteria for reinterventions, need for adjunctive treatment modalities or appropriate time for follow‐up.^8^ Furthermore, given poor clinical outcomes in patients with necrotizing pancreatitis and underlying DPDS, data on the impact of LAMS in this disease subset are lacking.

Therefore, given the increasing use, unique characteristics, and differences in the treatment approach when using LAMS compared to plastic stents,^4^, ^9^ the present study was conducted to compare clinical outcomes of patients treated in two time intervals by two different approaches: plastic stents versus integrating LAMS with selective use of plastic stents (Orlando Protocol). More importantly, a structured approach entitled the Orlando Protocol for endoscopic management of PFC was adopted within the treatment algorithm with the objective of standardizing outcomes in the new era of LAMS.

## Methods

The study included all consecutive patients with PFCs who underwent endoscopic treatment over a 10‐year period from January 2010 to August 2020. PFCs were categorized by consensus involving a dedicated radiologist and treating endoscopist according to the revised Atlanta classification (Appendix S1).^10^ Patients aged ≥18 years with current or previously documented pancreatitis and symptomatic PFCs who underwent endoscopic drainage using plastic or LAMS endoprostheses (AXIOS; Boston Scientific Corporation, Natick, MA, USA) were included. The exclusion criteria were patients with PFCs measuring <6 cm in size, irreversible thrombocytopenia (platelet count <50 × 10^9^/L) or coagulopathy (international normalized ratio >1.5), underlying malignancy, or unable to undergo anesthesia.

Data for first time interval (January 2010 to January 2015), when PFCs were treated by an algorithmic approach using plastic stents, were retrospectively analyzed from prospectively maintained databases at two tertiary medical centers – University of Alabama at Birmingham (January 2010 to June 2012; approval no. X100824005) and AdventHealth Orlando (July 2012 to January 2015; approval no. 741812). Data for the second time interval (February 2015 to August 2020) when PFCs were treated using a stepwise approach integrating LAMS with selective use of plastic stents, were retrospectively analyzed from a prospective registry comprising 292 data endpoints (approval no. 700323; clinicaltrials.gov registration no. NCT02422095). The registry was designed in 2015 when LAMS was first introduced and prospectively measured 10 specific endpoints that included the impact of integrated treatment approach on clinical outcomes. Study registries were previously queried to examine LAMS‐related adverse events, impact of endoprostheses on DPDS, and clinical outcomes in 53 patients with walled‐off necrosis.^4^, ^7^, ^9^, ^11^ Excluded from analysis were patients enrolled in randomized trials pertaining to PFC management.^12^, ^13^

All patients or their legally authorized representatives provided written informed consent for undergoing procedures and the study was approved by the institutional review boards of participating hospitals. As only standard of care was practiced, no study‐related interventions were undertaken, and the study involved analysis of prospective databases or registry, the need for patient consent for research was waived. All authors had access to study data and have reviewed and approved the final manuscript.

### Treatment protocol

Patients were divided into two time intervals according to the treatment approach (group 1, January 2010 to January 2015; group 2, February 2015 to August 2020). All patients underwent preprocedure contrast‐enhanced computed tomography (CT) of abdomen and pelvis when possible (unless absolutely contraindicated due to a history of anaphylactic reaction to iodine or severe renal failure) to determine PFC characteristics: extent, size, type (pseudocyst vs. walled‐off necrosis), proximity to stomach/duodenum and suitability for endoscopic drainage (encapsulation). Intravenous antibiotics were administered before the procedure and continued for 5 days. All transmural drainage procedures in both time intervals were undertaken using a therapeutic linear array echoendoscope. A percutaneous gastrojejunostomy or nasojejunal feeding tube was placed in patients unable to tolerate oral intake and 14‐Fr percutaneous catheters were inserted into areas of collection not accessible for endoscopic drainage.

#### Index intervention: plastic stent approach (January 2010 to January 2015)

Treatment was tailored to size and location of PFCs and a stepwise approach was adopted according to previously validated protocol (Fig. S1).^9^ Two 7‐Fr, 4 cm or 10‐Fr, 4 cm double pigtail plastic stents were then placed under endoscopic ultrasound‐guidance to facilitate transmural drainage using single‐gate (Video S1), multi‐gate or dual modality techniques. Additional technical details are provided in Appendix S1 and Table S1.

#### Index intervention: integrated LAMS approach – Orlando Protocol (February 2015 to August 2020)

**Figure 1:**
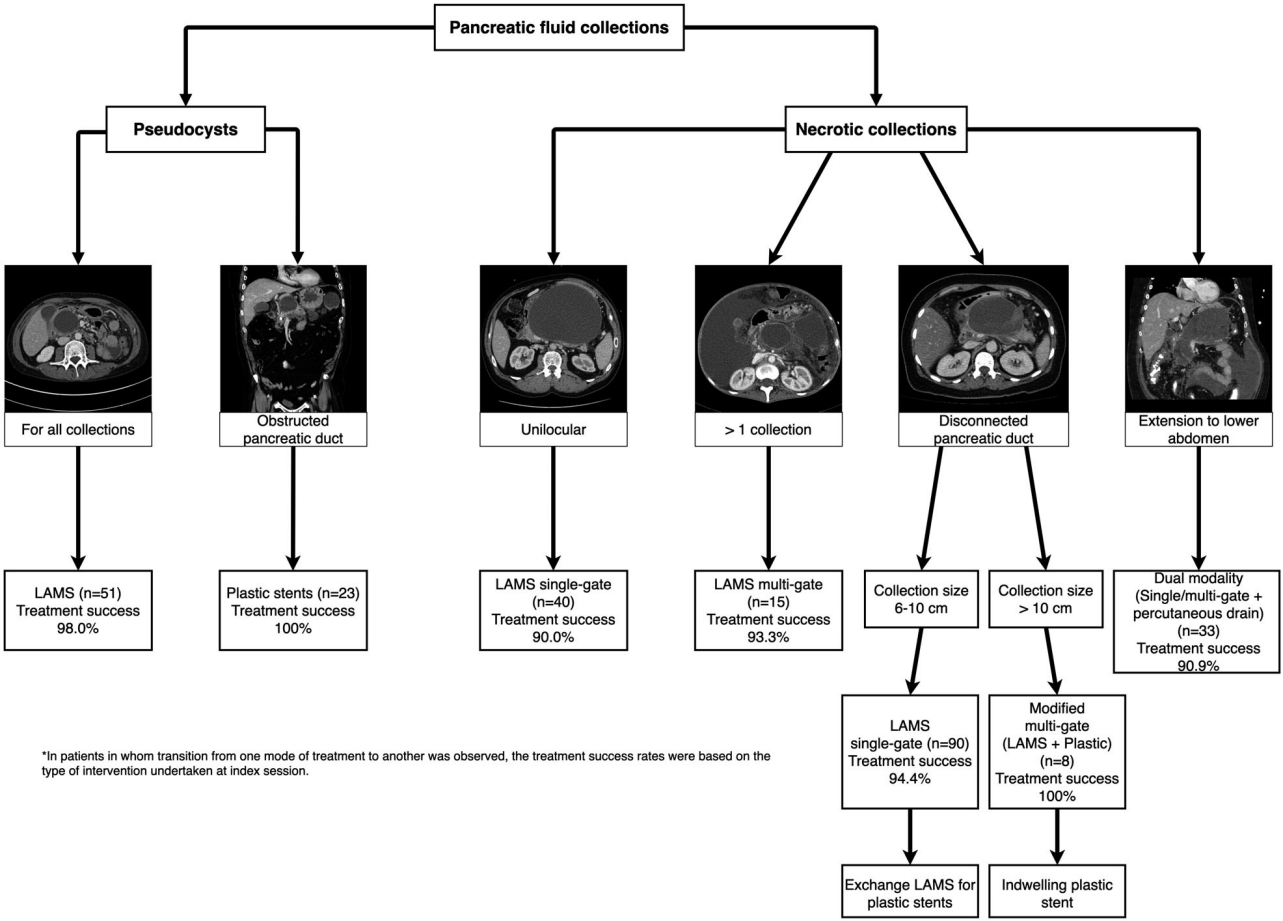
Integrated lumen‐apposing metal stents (LAMS) approach to endoscopic management of pancreatic fluid collections.

Drainage was undertaken using single‐gate (Video S2), multi‐gate (Video S3), modified multi‐gate (Video S4) or dual modality (Video S5) techniques based on the extent, size and location of PFC and pancreatic duct integrity (Fig. 1). Given the high risk for PFC recurrence, plastic stents (7‐Fr, 4 cm double pigtail) were selectively used for transmural drainage in patients with pseudocysts and obstructed pancreatic ducts (stones or strictures) that could not be treated successfully by endoscopic retrograde cholangiopancreatography (Video S6). Additional technical details are provided in Appendix S1 and Table S1.

#### Reinterventions for suboptimal response

In patients with pseudocysts and necrotic collections comprised predominantly of liquid debris, additional transmural tracts (using the original type of endoprosthesis) were created to facilitate better drainage. However, if necrotic cavity contained predominantly solid debris, direct endoscopic necrosectomy (DEN) was undertaken (Video S7). Further technical details, including drainage catheter management, are included in Appendix S1.

#### Treatment failure

A maximum of four interventions were performed per patient. If there was no clinical or radiological improvement, a consult was obtained to pursue surgical necrosectomy as definitive treatment. Emergent surgical consultation was also obtained for acute clinical deterioration during any phase of treatment.

#### Patient follow‐up

While patients with pseudocysts were discharged if clinical improvement was observed, a CT scan was obtained routinely at 72 h in patients with necrotic collections to assess treatment response. In patients with clinical and radiological improvement, percutaneous drainage catheters were removed as appropriate and patients were discharged from the hospital. If treatment response was suboptimal, endoscopic and/or percutaneous reinterventions were performed as outlined above and in Figure S2. Further details on outpatient follow‐up for the plastic stent cohort and integrated LAMS cohort (Video S8) are detailed in Appendix S1.

### Definitions

Treatment success was defined as reduction in PFC size to 2 cm or less on follow‐up CT in conjunction with symptom resolution at 6 months. Treatment failure was defined as persistence of PFC on follow‐up CT, need for surgical necrosectomy, disease‐related death or ongoing treatment of underlying disease at 6 months. Suboptimal treatment response was defined as lack of clinical improvement (resolution of systemic inflammatory response syndrome/sepsis)^14^ and less than 25% decrease in the size of PFC on CT at 72 h postintervention. Recurrence was defined as new occurrence of the PFC following initial treatment success within 6‐month follow‐up. Integrated LAMS approach was defined as patients who underwent PFC drainage between February 2015 and August 2020 using LAMS with selective application of plastic stents when indicated for transmural drainage and/or exchange of LAMS. Definitions of individual drainage techniques are stated in Appendix S1.

### Outcome measures

The primary outcome measure was treatment success, which was compared between two management approaches (plastic stents vs. integrated LAMS). The secondary outcome measures were rate of recurrence, reinterventions, rescue surgery, adverse events, and duration of hospitalization.

### Sample size and statistical analysis

Details on sample size calculation, statistical analysis and logistic regression are included in Appendix S1.

## Results

### Baseline characteristics

A total of 387 patients were enrolled, 160 in the plastic group and 227 in the integrated LAMS group (Table 1). While a significantly higher proportion of patients were females in the plastic stent cohort, the integrated LAMS cohort comprised of more patients with alcoholic pancreatitis, multiple PFCs and necrotic collections. In both cohorts, DPDS and infection was observed in >50% and 25% of patients, respectively.

**Table 1:** Patient details and pancreatic fluid collection characteristics

	Plastic (*n* = 160)	Integrated LAMS (*n* = 227)	*P*‐value
	
Age (years)			
Mean (SD)	51.3 (16.9)	53.8 (14.3)	–
Median	53	55	0.261
IQR	39–64	43–64	
Sex, *n* (%)			
Female	78 (48.8)	75 (33.0)	0.002
Male	82 (51.2)	152 (67.0)	–
Race, *n* (%)			
White	131 (81.9)	190 (83.7)	0.277
Black	18 (11.2)	16 (7.0)	–
Other	11 (6.9)	21 (9.3)	–
Etiology of pancreatitis, *n* (%)			
Alcohol	39 (24.4)	97 (42.7)	<0.001
Gallstones	32 (20.0)	45 (19.8)	–
Idiopathic	45 (28.1)	58 (25.6)	–
Other^†^	44 (27.5)	27 (11.9)	–
Serum white cell count (×10^9^/L)			
Mean (SD)	12.5 (6.3)	11.2 (5.6)	–
Median	9.2	9.6	0.060
IQR	7.7–17.8	7.2–14.6	–
Serum albumin (g/dL)			
Mean (SD)	3.0 (0.79)	3.1 (0.67)	–
Median	3.0	3.1	0.211
IQR	2.2–3.7	2.6–3.6	–
CT severity index^‡^			
0–2	27 (16.9)	42 (18.5)	0.680
4–6	90 (56.2)	79 (34.8)	–
8–10	43 (26.9)	106 (46.7)	–
PFC type, *n* (%)			
Acute PFC	13 (8.1)	4 (1.8)	0.002
Pseudocyst	64 (40.0)	70 (30.8)	–
Acute necrotic collection	4 (2.5)	27 (11.9)	–
WON	79 (49.4)	126 (55.5)	–
Duration of PFC (weeks)			
Mean (SD)	10.1 (10.2)	8.0 (7.9)	0.035
Median	8	5	–
IQR	4.5–12	4–10	–
Size of PFC (cm)			
Mean (SD)	10.3 (4.5)	10.6 (5.1)	0.947
Median	9.0	9.5	–
IQR	7.0–12.3	6.4–13.4	–
Location of PFC, *n* (%)			
Head/uncinate	20 (12.5)	32 (14.1)	0.650
Neck/body/tail	140 (87.5)	195 (85.9)	–
Infected PFC, *n* (%)	41 (25.6)	57 (25.1)	0.909
Multiple PFC, *n* (%)	25 (15.6)	65 (28.6)	0.003
DPDS present, *n* (%)			
Yes	81 (50.6)	121 (53.3)^¶^	0.603
No^§^	51 (31.9)	81 (35.7)	–
Unknown	28 (17.5)	25 (11.0)	–

^†^ Plastic group: pancreas divisum (*n* = 1), medication‐induced (*n* = 2), post‐ERCP (*n* = 4), post‐surgical/trauma (*n* = 31), hypertriglyceridemia (*n* = 6). Integrated LAMS group: pancreas divisum (*n* = 1), medication‐induced (*n* = 2), post‐ERCP (*n* = 1), post‐surgical/trauma (*n* = 12), hypertriglyceridemia (*n* = 11).

^‡^ CT severity index is a tool for estimating the radiological severity of pancreatitis and the score generated by combining the degree of inflammation, degree of necrosis and presence of extrapancreatic complications. CT severity index of 0–2 corresponds to mild pancreatitis, 4–6 to moderate pancreatitis and 8–10 to severe pancreatitis.^18^

^§^ Of 45 patients with pancreatic duct leak at ERCP, pancreatic stents were successfully placed for pancreatic duct leak in 31 patients in plastic group and seven patients in integrated LAMS group (84.4%). Pancreatic duct stents were not placed in any patients with DPDS.

^¶^ DPDS was diagnosed using magnetic resonance cholangiopancreatography in 17 patients and with ERCP in 104 patients.

CT, computed tomography; DPDS, disconnected pancreatic duct syndrome; ERCP, endoscopic retrograde cholangiopancreatography; IQR, interquartile range; LAMS, lumen‐apposing metal stent; PFC, pancreatic fluid collection; SD, standard deviation; WON, walled‐off necrosis.

### Procedure details

A significantly higher proportion of patients who underwent plastic stent placement in both cohorts had 7‐Fr as compared to 10‐Fr endoprostheses placed (Table 2). Also, more patients underwent multi‐gate drainage in the plastic stent cohort as compared to integrated LAMS cohort. 15% of patients in both cohorts underwent dual modality drainage and one‐third of patients in both cohorts underwent enteral feeding tube placement. In the integrated LAMS cohort, exchange of LAMS for plastic stents in patients with DPDS was unsuccessful in 28 patients (23.1%) as the cavity had sealed‐off completely at follow‐up endoscopy.

**Table 2:** Procedure details

	Plastic (*n* = 160)	Integrated LAMS (*n* = 227)	*P*‐value
	
Route of drainage, *n* (%)			
Transesophageal	3 (1.9)	0	0.111
Transgastric	144 (90.0)	206 (90.7)	–
Transduodenal	13 (8.1)	21 (9.3)	–
Stent size and type, *n* (%)			
Plastic			
7‐Fr	139 (86.9)	14 (6.2)	<0.001
10‐Fr	21 (13.1)	0	–
LAMS			
15 mm	0	153 (67.4)	–
20 mm	0	60 (26.4)	–
Single‐gate technique, *n* (%)^†^	123 (76.9)	204 (89.9)	0.001
Multi‐gate technique, *n* (%)	37 (23.1)	23 (10.1)^‡^	0.001
Dual modality technique, *n* (%)	24 (15.0)^§^	33 (14.5)	0.899
Procedure duration (min)			
Mean (SD)	30.0 (21.6)	19.5 (12.9)	–
Median	22	16	0.001
IQR	15–33.5	9–28	–
Nutrition via enteral feeding tube, *n* (%)	58 (36.3)	77 (33.9)	0.636

^†^ Endoscopic necrosectomy was performed at index procedure in five patients in the plastic cohort and in seven patients in the integrated LAMS cohort.

^‡^ Multi‐gate technique in the integrated LAMS group were using only LAMS in 15 patients and modified multi‐gate technique (using both LAMS and plastic stents) in eight patients.

^§^ Nasocystic drains were inserted in 17 patients and percutaneous drains were inserted in seven patients.

IQR, interquartile range; LAMS, lumen‐apposing metal stent; SD, standard deviation.

### Treatment outcomes

Treatment success was significantly higher for integrated LAMS as compared to plastic stent approach at 95.6% vs. 89.4% (*P* = 0.018) for all PFC types and was also significantly higher in necrotic collections (94.1 vs. 84.3, *P* = 0.014; Table 3, Table S2). A significantly higher proportion of patients treated using the plastic stent approach required rescue surgery as compared to the integrated LAMS approach (7.5 vs. 1.3%, *P* = 0.002). Treatment failure in the plastic cohort were managed by surgery in 12 patients, conservative measures in five and resulted in death in eight patients; treatment failure in the integrated LAMS cohort was managed by surgery in three patients, conservative measures in seven and resulted in deaths in six patients. There was no significant difference in the rates of all‐cause mortality or adverse events between treatment approaches (Table 3, Table S3). 25% of patients in both cohorts required reinterventions that included additional drainage or DEN and there was no significant difference in rate of PFC recurrence at 6‐month follow‐up (2.5 vs. 3.1%, *P* = 0.99). In the plastic cohort, PFC recurrence was observed in four patients, all of whom had DPDS, and were managed by endoscopic techniques in three and surgery in one. In the integrated LAMS cohort, PFC recurrence was observed in seven patients, with DPDS present in all seven of these patients. One patient with DPDS had disease recurrence despite an indwelling plastic stent, likely due to stent dysfunction; in six remaining patients with DPDS, exchange of LAMS for plastic stents was unsuccessful due to collapse of the PFC cavity at follow‐up.

**Table 3:** Summary of treatment outcomes for all PFC types

	Plastic (*n* = 160)	Integrated LAMS (*n* = 227)	*P*‐value
	
Technical success, *n* (%)^†^	159 (99.4)	227 (100)	0.413
Treatment success, *n* (%)^‡^	143 (89.4)	217 (95.6)^§^	0.018
Recurrence, *n* (%)	4 (2.5)^¶^	7 (3.1)^††^	0.999
Reinterventions encompassing necrosectomy/additional endoscopic drainage, *n* (%)	40 (25.0)	58 (25.6)	0.902
Total no. of interventions			
Mean (SD)	1.4 (0.70)	1.5 (1.0)	–
Median	1	1	0.627
IQR	1–1.5	1–2	–
Rescue surgery, *n* (%)	12 (7.5)	3 (1.3)	0.002
Adverse events, *n* (%)	34 (21.3)	47 (20.7)	0.897
Mortality, *n* (%)			
All‐cause mortality	13 (8.1)	17 (7.5)	0.818
From underlying disease/intervention	8 (5.0)	6 (2.6)	0.221
Duration of hospital stay (days)			
Mean (SD)	8.5 (17.8)	8.1 (10.2)	0.774
Median	2	4	–
IQR	1–8	1–11	–

^†^ Technical failure was encountered in one patient in the plastic group ‐ in this patient, gastric perforation occurred during cystogastrostomy and required surgical intervention.

^‡^ Treatment failure in the plastic cohort was encountered in 17 patients, which was managed by surgery in 12 patients, managed conservatively in five patients and resulted in deaths in eight patients. Treatment failure in the integrated LAMS group was encountered in 10 patients, which was managed by surgery in three patients, managed conservatively in seven patients and resulted in deaths in six patients.

^§^ There was no significant difference in treatment success rates between 15 mm diameter and 20 mm diameter LAMS (96.1% vs. 93.3%, *P* = 0.473).

^¶^ Recurrence occurred in four patients in the plastic cohort and all four of these patients had underlying DPDS. In these patients, recurrence was managed with surgery in one patient and repeat endoscopic drainage in three patients.

^††^ Recurrence occurred in seven patients in the integrated LAMS cohort and all of these seven patients had underlying DPDS. Indwelling plastic stents were present in one patient, however presumably was dysfunctional; in the remaining six patients, replacement of LAMS with plastic stents was not successful. Four of seven patients with PFC recurrence underwent repeat endoscopic ultrasound‐guided drainage with placement of plastic endoprostheses and the remaining three patients in whom the collections measured 3–5 cm in size were managed conservatively.

DPDS, disconnected pancreatic duct syndrome; IQR, interquartile range; LAMS, lumen‐apposing metal stent; PFC, pancreatic fluid collection; SD, standard deviation.

### Predictors of treatment success

On multivariate logistic regression analysis (Table 4), management by integrated LAMS approach was the only factor associated with treatment success after adjustment for patient demographics, disease, PFC characteristics and DPDS status (odds ratio 2.7, 95% confidence interval 1.1–6.4; *P* = 0.028).

**Table 4:** Multivariable logistic regression analysis to identify factors associated with treatment success

Variable	Odds ratio	95% CI	*P*‐value
Treatment period: integrated LAMS vs. plastic	2.7	1.1–6.4	0.028
Age: ≤50 vs. >50 years	0.90	0.38–2.1	0.814
Sex: male vs. female	0.82	0.34–1.9	0.650
Ethnicity: Caucasian vs. non‐Caucasian	0.82	0.26–2.6	0.740
Etiology: gallstones/alcohol vs. other	1.4	0.61–3.3	0.421
CT severity index: <8 vs. ≥8	0.90	0.34–2.4	0.832
PFC type: pseudocyst vs. necrotic collection	1.2	0.40–3.5	0.753
Size of PFC: <10 cm vs. ≥10 cm	1.4	0.54–3.4	0.521
Duration of PFC: >6 weeks vs. ≤6 weeks	1.7	0.73–4.0	0.214
Multiple PFCs: no vs. yes	1.5	0.58–3.9	0.403
Location of PFC: neck/body/tail vs. head/uncinate	0.33	0.04–2.7	0.300
Disconnected pancreatic duct: no vs. yes	0.94	0.35–2.5	0.894

CI, confidence interval; CT, computed tomography; LAMS, lumen‐apposing metal stent; PFC, pancreatic fluid collection.

## Discussion

Endoscopic techniques to treat PFCs have evolved over the past decade. Randomized trials suggest that endoscopic approaches may be comparable or even superior to minimally invasive surgery with decreased rate of postoperative adverse events, lower long‐term morbidity, shorter length of hospital stay and is less costly.^13^, ^15^, ^16^ While endoprosthesis placement is a critical first step, it is only the first of several subsequent steps in endoscopic management. Other critical issues include tailoring procedural technique to radiological findings, criteria and timing for reinterventions, optimizing treatment strategy to minimize PFC recurrence and scheduling timely follow‐up to ensure optimal outcomes. However, outside of clinical trial settings, none of these steps have been evaluated in a systematic manner thereby resulting in poor standardization of treatment approaches. The proposed Orlando Protocol, by comparing outcomes between a traditional (plastic stent) versus more recent (integrated LAMS) treatment approach, provides new and valuable information that may help standardize management. The new approach demonstrated a treatment success rate of 95% in this challenging patient population.

Firstly, we demonstrated a small but definitive incremental improvement in treatment success for the integrated LAMS approach as compared to the plastic stent approach. This tangible improvement in outcome however, is unlikely to be related solely to type of endoprosthesis used, but to the overall treatment approach when using LAMS. As shown in prior investigations, the procedural duration for index interventions when using LAMS was significantly shorter. This is an important attribute as patients are generally critically ill at presentation and cannot endure prolonged procedures.^11^, ^16^ Also, we observed that by facilitating better drainage, the integrated LAMS approach eliminated need for nasocystic catheters which are prone to frequent dysfunction and are a source of discomfort to patients. The method also minimized need for the multi‐gate technique in phase II, which was otherwise critical to augment drainage in the plastic stent cohort. Additionally, by serving as an easy conduit to perform DEN, the need for rescue surgery appeared to be relatively low when using LAMS. Secondly, there is growing awareness that DPDS can negatively impact treatment outcomes as patients are sicker and PFC recurrence rates are higher as compared to patients with intact pancreatic duct.^11^ By tailoring management protocol to this patient subset, we believe that the integrated LAMS approach reduces disease morbidity by facilitating better drainage in the early phase and reducing PFC recurrence in later phase. Thirdly, despite increasing popularity of LAMS, we identified three distinct clinical settings where use of plastic stents remain relevant – patients with pseudocysts in setting of obstructive pancreatic duct, modified multi‐gate drainage in patients with large necrotic collections and underlying DPDS, and to exchange for LAMS in patients diagnosed with DPDS at follow‐up. Fourthly, unlike in previous studies,^12^, ^17^ no significant difference in the rate of adverse events was observed between the plastic stent approach and the integrated LAMS approach. We believe that this was due to the LAMS being removed within 3–4 weeks post‐index intervention in our patient cohort given risks of stent‐related adverse events in patients with delayed removal. Finally, to our knowledge, this study is the first to provide a comprehensive framework for endoscopic management of PFCs where a predefined treatment strategy was validated in a large cohort of patients in a clinical setting. The study incorporated state‐of‐the‐art procedural techniques and treatment modalities that included objective criteria for index intervention, reinterventions and follow‐up. Therefore, we believe that the present study provides a robust framework for establishment of future treatment strategies and clinical trial protocols.

This study has several limitations. First, reported results derive from a center with advanced expertise in pancreatic‐biliary disorders. Several aspects of treatment, such as timing/use of endoscopic retrograde cholangiopancreatography to assess pancreatic duct, placement of percutaneous or nasocystic catheters for lavage, timing for interventions, indication for rescue surgery, were multi‐disciplinary and institution specific. Second, although we attempted to tailor interventions to most clinical presentations, challenges persist. In patients with DPDS and small collections, exchange of LAMS for plastic stents was not uniformly successful as the cavity can seal off resulting in PFC recurrence at long‐term. This clinical conundrum remains to be addressed. Third, as a proportion of patients with PFCs can be managed without intervention and the study does not include patients treated by other modalities, the findings may not be universally applicable to all PFC patients. Fourth, magnetic resonance imaging, which is considered superior to CT for evaluation of necrotic collections, was not performed in all patients. Fifth, although we utilized LAMS for patients with uncomplicated pseudocysts due to the technical ease of placement, plastic stents are equally effective.^12^ Sixth, while it is possible to speculate that increasing experience or the type of endoprosthesis alone may have contributed to observed outcomes, this is less likely. By exclusion of subjects performed prior to 2010, adoption of standard‐of‐care techniques in both phases that are still currently relevant, involvement of only experienced operators in phase I (S.V., R.H., C.M.W.), performance of procedures by including more recently trained interventionalists in phase II (J.Y.B., U.N.), and standardizing management protocols in both phases, we believe that observed outcomes, treatment success of 95.6% in routine clinical setting, was due to the integrated LAMS approach. Also, in a prior randomized trial that compared LAMS and plastic stents, we demonstrated no significant difference in clinical outcomes.^12^

In conclusion, by yielding an overall treatment success of 95%, the proposed management integrating LAMS with selective use of plastic stents was found to be superior to an approach using only plastic stents in patients with symptomatic PFCs.

## Conflict of Interest

J.Y.B. is a consultant for Boston Scientific Corporation and Olympus America Inc. S.V. is a consultant for Boston Scientific Corporation, Olympus America Inc., Covidien and Creo Medical. R.H. is a consultant for Boston Scientific Corporation, Olympus America Inc., Covidien, Creo Medical, Nine Points Medical and Cook Medical. The other authors declare no conflict of interest for this article.

## Funding Information

None.

## Supporting information


**Figure S1** Algorithmic approach to management of pancreatic fluid collections using plastic stent approach.

**Figure S2** Structured approach to reinterventions for suboptimal clinical response.

**Table S1** Summary of key differences in treatment approaches in time intervals.
**Table S2** Summary of treatment outcomes for necrotic collections only.
**Table S3** Disease and procedure‐related adverse events.
**Appendix S1** Methods.

**Video S1** Endoscopic ultrasound‐guided placement of plastic stents.

**Video S2** Site selection for endoscopic ultrasound‐guided LAMS placement.

**Video S3** Multi‐gate drainage technique using LAMS in patients with noncommunicating PFCs.

**Video S4** Modified multi‐gate technique in patients with underlying DPDS and necrotic collections measuring more than 10cm in size.

**Video S5** Dual modality drainage technique for PFCs tracking to the lower abdomen.

**Video S6** Endoscopic ultrasound‐guided drainage of pseudocyst using plastic stent in patient with obstructive main pancreatic duct after failed endoscopic retrograde cholangiopancreatography.

**Video S7** Direct endoscopic necrosectomy.

**Video S8** Exchange of LAMS for plastic endoprosthesis at follow‐up in patients with underlying DPDS.

**Video S9** Clearance of debris from within the cavity as visualized by endoscopic ultrasound where a change from heterogeneity to anechogenicity is observed.
