# Supplementary figures and images for: Validation of the Orlando Protocol for endoscopic management of pancreatic fluid collections in the era of lumen‐apposing metal stents
Source: Dig Endosc. 2021 Sep 6;34(3):612–21. doi: 10.1111/den.14099 (PMC9290475; doi:10.1111/den.14099)

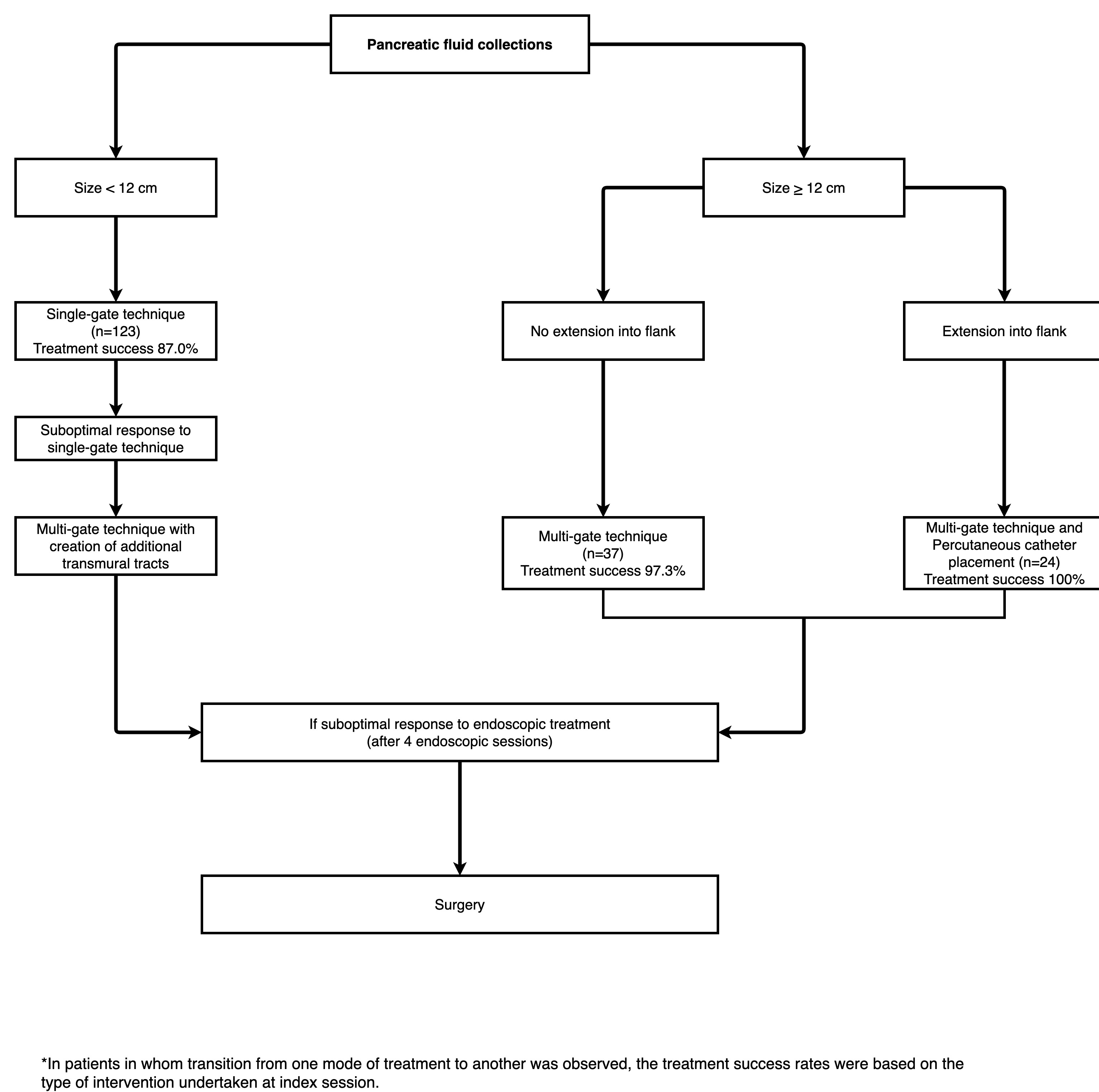

Supplement: Supplementary file 1 — Figure S1 Algorithmic approach to management of pancreatic fluid collections using plastic stent approach. [file DEN-34-612-s004.jpg]

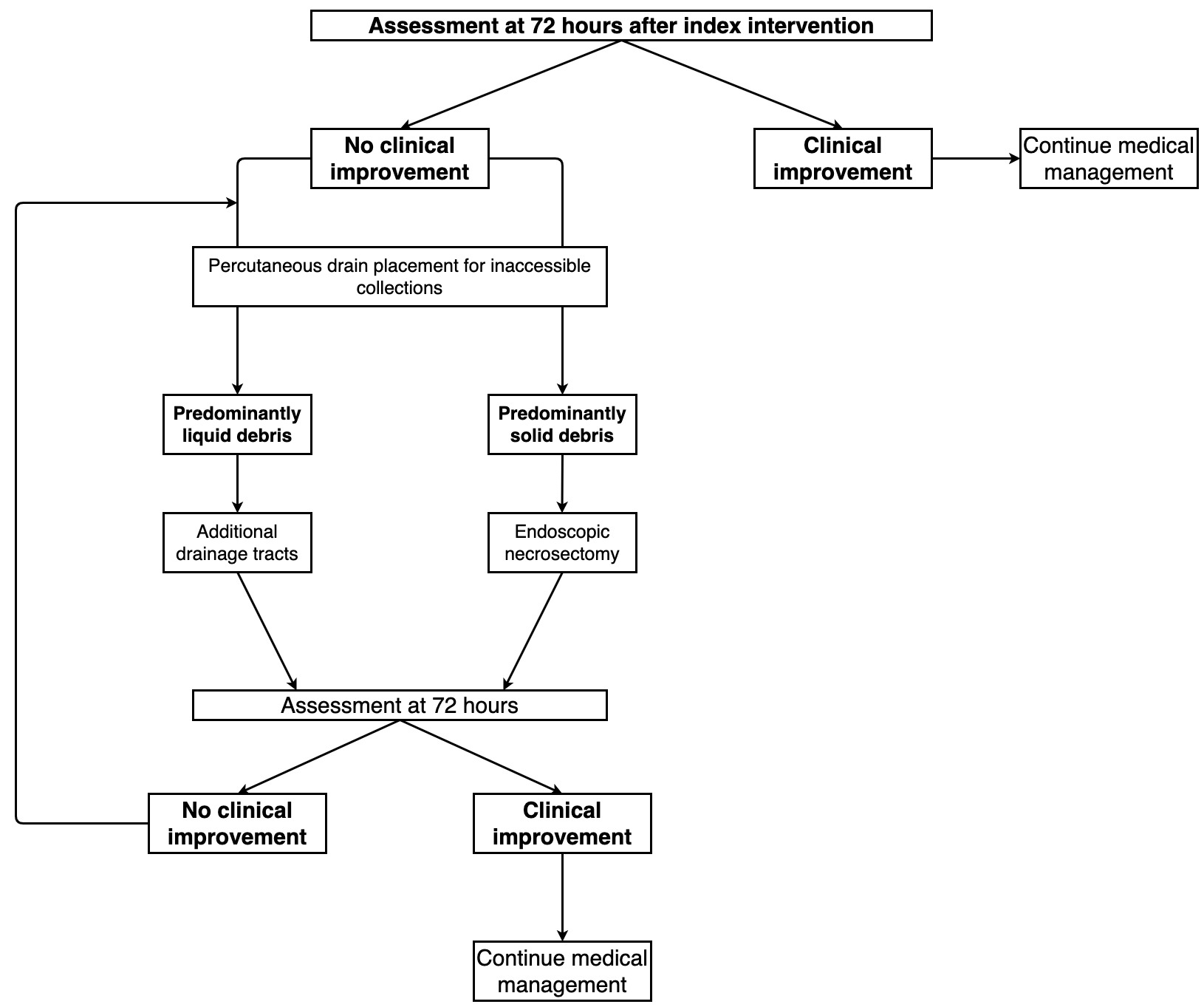

Supplement: Supplementary file 2 — Figure S2 Structured approach to reinterventions for suboptimal clinical response. [file DEN-34-612-s012.jpg]
