# Supplementary material for: Validation of the Orlando Protocol for endoscopic management of pancreatic fluid collections in the era of lumen‐apposing metal stents
Source: Dig Endosc. 2021 Sep 6;34(3):612–21. doi: 10.1111/den.14099 (PMC9290475; doi:10.1111/den.14099)
Supplement: Supplementary file 3 — Table S1 Summary of key differences in treatment approaches in time intervals. Table S2 Summary of treatment outcomes for necrotic collections only. Table S3 Disease and procedure‐related adverse events. Appendix S1 Methods. [file DEN-34-612-s009.docx]

**SUPPLEMENTAL FILE to manuscript:**

**Validation of the Orlando Protocol for endoscopic management of pancreatic fluid collections in the era of LAMS**

Ji Young Bang MD, MPH, C. Mel Wilcox MD, Juan Pablo Arnoletti MD, Shajan Peter MD,

John Christein MD, Udayakumar Navaneethan MD, Robert Hawes MD, Shyam Varadarajulu, MD

**Appendix S1 Methods**

**Treatment protocol**

Pancreatic fluid collections were diagnosed in patients with a history of pancreatitis who underwent CT imaging. In these patient, the pancreatic fluid collections were categorized according to the presence of necrotic debris and timing of pancreatitis. Pancreatic fluid collections without necrotic debris (solid material) were categorized as acute peripancreatic fluid collections if the onset of pancreatitis was < 4 weeks and as pancreatic pseudocysts if the onset of pancreatitis was ≥ 4 weeks. Collections containing solid necrotic material were categorized as acute necrotic collections if the onset of pancreatitis was < 4 weeks and as walled-off necrosis if the onset of pancreatitis was ≥ 4 weeks.

At index intervention in both cohorts, after transluminal stent placement, the PFC was irrigated with normal saline and continued until the efflux revealed no solid debris. This was performed by attaching a tubing from the water pump (Olympus OFP-2, Center Valley, Pennsylvania, USA) to an ERCP cannula passed through the echoendoscope or a previously placed percutaneous drain. The clearance of debris from within the cavity was also confirmed endosonographically by a change in the echogenicity of the PFC from heteroechogenicity to anechogenicity (Supplemental Video 9).

Necrosectomy was not performed at index intervention unless there was no drainage of necrotic contents after endoprosthesis placement. This required dilation of the transmural tract to 15-18mm in patients undergoing placement of plastic endoprosthesis or 15mm diameter LAMS; the necrotic cavity was intubated directly via the stent lumen without dilation, in patients treated with 20mm diameter LAMS.

**Index intervention: Plastic stent approach (January 2010 to January 2015)**

Prior to undertaking transmural drainage, if DPDS had not been demonstrated on CT or magnetic resonance cholangiopancreatography (MRCP), the patient underwent endoscopic retrograde cholangiopancreatography (ERCP) to assess for pancreatic duct integrity and placement of a bridging stent if a leak was observed.

For all pseudocysts (irrespective of the size) and WON <12 cm in size, the PFC was punctured with a 19G fine needle aspiration (FNA) needle to gain access and a 0.025-inch guidewire was coiled within the PFC cavity. The transmural tract was then dilated to 12 to 15mm using a radial expansion balloon (CRE™; Boston Scientific, Natick, Massachusetts, USA) over the guidewire and two 7Fr, 4cm or 10Fr, 4cm double pigtail stents (Cook Endoscopy, Winston‐Salem, North Carolina, USA) were deployed sequentially (Supplemental Video 1). If the collection was necrotic, a 7Fr nasocystic catheter was also inserted for lavage of the necrotic cavity with 200-250mL of normal saline every 4 to 6 hours.

For multiple non-communicating PFCs ≥6cm in size or WON >12cm (irrespective of ductal integrity), additional internal conduits were created using the multi-gate technique. For necrotic collections with extension to the flank(s), a 14Fr percutaneous catheter was inserted under CT-guidance into the dependent portion of the collection (dual modality technique).

**Index intervention: Integrated LAMS approach (February 2015 to August 2020)**

A single LAMS was placed in all pseudocysts with patent pancreatic duct and unilocular necrotic collections (single-gate technique). While the majority of procedures were performed using 15mm diameter LAMS (AXIOS stent, Boston Scientific Corporation, Natick, Massachusetts), given the likelihood of performing endoscopic necrosectomy, a 20mm diameter LAMS was used when more than one-third (33%) of the collection comprised of solid debris. LAMS with 15mm saddle length were used for drainage of PFCs that were located >10mm from the gastrointestinal lumen; 10mm saddle length LAMS were used in all other cases. At deployment, every attempt was made to position the endoprosthesis in the distal stomach (gastric body or antrum rather than the cardia), in order to facilitate easy access to the necrotic cavity for performing direct endoscopic necrosectomy (DEN) (Supplemental Video 2).

Given the high risk for PFC recurrence, plastic stents (7Fr, 4cm double pigtail) were selectively used for transmural drainage in patients with pseudocysts and obstructed pancreatic ducts (stones or strictures) that could not be treated successfully by ERCP (Supplemental Video 6). The endoprostheses were left in situ indefinitely to minimize the risk of PFC recurrence. In patients with multiple non-communicating collections, one LAMS was placed into each collection ≥6 cm in size (the multi-gate technique).

In patients with DPDS and WON measuring >10cm in size, LAMS was placed first into one tract in the proximal most part of the collection (which usually corresponded to the gastric body/antrum) and two double pigtail plastic endoprostheses were then placed into a second tract located more distally towards the pancreatic tail (which usually corresponded to the proximal stomach/gastric cardia). This “modified” multi-gate strategy was adopted to facilitate better drainage given the suboptimal clinical response to conventional single tract drainage in patients with underlying DPDS and large necrotic collections. As patients with PFC in the setting of DPDS frequently require an endoscopic necrosectomy, positioning LAMS in the gastric body/antrum, as opposed to the gastric cardia, enabled easier access to the necrotic cavity for performing debridement. Also, as patients with DPDS have high rates of PFC recurrence, only the LAMS was removed at 3-4-week follow-up and plastic stents were left indwelling indefinitely to drain the disconnected segment. By placing two different types of endoprostheses in this patient subset, the LAMS tract guaranteed access for potential necrosectomy and the indwelling plastic endoprosthesis served to minimize PFC recurrence in the long-term.

For WON with extension to the flank(s), in addition to LAMS placement, a 14‐Fr percutaneous catheter was inserted into the dependent portion of the necrotic collection for irrigation and drainage. The percutaneous catheters were flushed with 200-250mL of normal saline every 4-6 hours until necrotic debris were no longer visible on lavage.

**Reinterventions for suboptimal response**

***Pseudocysts***

In both plastic stent and integrated LAMS cohorts, additional transmural drainage tracts were created if the residual fluid collection was ≥6cm in size. Otherwise, irrigation and lavage were performed by placing an ERCP catheter through the transmural tract and into the pseudocyst cavity to facilitate better drainage.

***Necrotic collections***

In patients with necrotic collections comprised predominantly of liquid debris, additional transmural tracts (using the original type of endoprosthesis) were created to facilitate better drainage. However, if the cavity contained predominantly solid debris, DEN was undertaken (Supplemental Video 7).

In the plastic stent cohort, performing endoscopic necrosectomy involved the removal of the plastic endoprostheses, dilation of the transmural tract to 15mm using a radial expansion balloon to facilitate the insertion of the gastroscope into the necrotic cavity. In the LAMS cohort, the gastroscope was advanced directly through the lumen of the endoprosthesis and into the necrotic cavity to undertake endoscopic necrosectomy. In patients with percutaneous catheters, the percutaneous tract was dilated to 48Fr using Savary dilators (Savary Gillard, Cook Endoscopy, Winston Salem, North Carolina, USA) and a fully-covered self‐expanding esophageal metal stent was placed with the proximal end of the stent located within the necrotic cavity and the distal end at the anterior abdominal wall. The esophageal stent was then used as a conduit to perform necrosectomy using a gastroscope. Endoscopic sessions were continued until at least 90% of the cavity comprised of red granulation tissue.

**Patient follow-up**

*Plastic stent cohort:* All patients underwent outpatient CT of abdomen/pelvis at 8-week follow-up. If the PFC had resolved and the main pancreatic duct was intact as observed on ERCP, the transmural stents were removed. In patients with DPDS or pancreatic duct obstruction by stones/strictures, two transmural stents were left indwelling to minimize the risk of PFC recurrence.

*Integrated LAMS approach:* Given the risk of delayed bleeding in patients treated with LAMS,^1^ outpatient CT of abdomen/pelvis was obtained at 3 to 4-week follow-up. If the PFC had resolved and the main pancreatic duct was intact at ERCP/MRCP, LAMS was removed. In patients with DPDS treated using single LAMS, the endoprosthesis was exchanged for two 7Fr double pigtail plastic stents (Supplemental Video 8).

In both treatment groups, if pancreatic duct leak was identified at ERCP, a transpapillary pancreatic duct stent was placed to bridge the leak. The drainage catheters were discontinued when follow-up CT demonstrated no residual fluid collection with less than 10ml discharge over a 72-hour period. Enteral nutrition was discontinued at follow‐up in all patients.

**Definitions**

Single-gate technique included endoscopic placement of LAMS or two plastic stents via a single tract. Multi-gate technique involved the placement of endoprosthesis via more than one tract. Modified multi-gate involved placement of LAMS in the distal stomach and two plastic stents via the proximal stomach to drain PFC >10cm in size in patients with underlying DPDS. Dual modality included the placement of 14Fr percutaneous drain (under CT-guidance) in conjunction with endoscopic transmural drainage.

**Sample size calculation**

A two-sided sample size calculation was performed based on the rate of treatment success, which was estimated at 95% for the LAMS-based approach and 85% for the plastic stent approach.^1-4^ A sample size calculation to detect a 10% difference in treatment success at 85% power and type I error (α) of 0.05 resulted in sample size estimation of 158 patients per group (PASS 15 Power Analysis and Sample Size Software, NCSS, LLC, Kaysville, Utah, USA).

**Statistical analysis**

Patient demographics, disease characteristics, procedural details and treatment outcomes were summarized and compared between groups. Continuous data were summarized as means with standard deviation and median with interquartile range and were compared using the Student’s t-test or Wilcoxon rank-sum test as indicated. Categorial data were summarized as frequencies with percentages and compared using chi-square or Fisher’s exact test as indicated.

In order to identify factors associated with treatment success, multiple logistic regression analysis was performed. Predictor variables of clinical importance or statistical significance were utilized in the regression analyses. Statistical significance was determined at p<0.05 and two-sided p-values were reported for comparison of outcome measures. Statistical analyses were performed using Stata 14 (Stata Corp, College Station, TX).

**References**

1. Bang JY, Navaneethan U, Hasan MK et al. Non-superiority of lumen-apposing metal stents over plastic stents for drainage of walled-off necrosis in a randomised trial. Gut 2019;68:1200-09.
2. Bang JY, Hasan MK, Navaneethan U, et al. Lumen-apposing metal stents for drainage of pancreatic fluid collections: when and for whom? Dig Endosc 2017;29:83-90.
3. Bang JY, Holt BA, Hawes RH, et al. Outcomes after implementing a tailored endoscopic step‐up approach to walled‐off necrosis in acute pancreatitis. Br J Surg 2014;13:1729-38.
4. Bang JY, Arnoletti JP, Holt BA et al. An endoscopic transluminal approach, compared with minimally invasive surgery, reduces complications and costs for patients with necrotizing pancreatitis. Gastroenterology 2019;156:1027-40.

**Table S1.** A summary of the key differences between the two treatment approaches

| **Assessment of pancreatic duct integrity**   - *Plastic stent approach:* At index intervention, an ERCP was performed prior to PFC drainage to assess for pancreatic duct integrity. If DPDS was observed, the plastic stents were left indwelling. If a leak was seen but with upstream opacification of the duct, a transpapillary stent was placed to bridge the leak. - *Integrated LAMS approach*: If an MRCP was not obtained, then an ERCP was performed at first follow-up, at 3 to 4 weeks after the index intervention. If DPDS was seen, LAMS was exchanged for double pigtail plastic stents, which were left indwelling. If a leak was seen but with upstream opacification of the duct, a transpapillary stent was placed to bridge the leak. - *Rationale:* Unlike plastic stents, LAMS cannot be left to indwell, and all patients require follow-up endoscopy for endoprosthesis removal. Consequently, assessment of ductal status was performed at follow-up in the integrated LAMS cohort. Also, more importantly, the presence of a fluid collection at index intervention limits the accuracy of MRCP in ascertaining ductal integrity.   **Procedural technique**   - *Plastic stent approach:* For all pseudocysts and WON <12 in size, a single tract was created for plastic stent placement. For multiple noncommunicating PFCs ≥6cm or WON >12 cm in size, additional transmural internal conduits were created to facilitate better drainage. - *Integrated LAMS approach:* A single LAMS was placed to drain unilocular PFC of any size (single-gate technique). For patients with multiple noncommunicating (discrete) collections ≥6cm in size, one LAMS was placed into each collection (multi-gate technique). In patients with DPDS in whom the necrotic collection measured >10cm in size, one LAMS and two plastic stents were deployed into two different tracts (modified multi-gate technique). In patients with DPDS in whom the WON measured <10 cm in size, drainage was accomplished using one LAMS (single-gate technique). In patients with fluid collections tracking to the lower abdomen, a 14Fr percutaneous drain was preferably placed under CT-guidance first followed by endoscopic transmural drainage (dual modality technique). The percutaneous catheters were capped following placement until endoscopic drainage was accomplished to prevent collapse of the cavity.   Given the high risk for PFC recurrence in patients with pseudocysts and obstructed pancreatic duct, two plastic stents (7Fr, 4cm, double pigtail) were selectively placed for transmural drainage at index intervention so that they can be left to indwell permanently.   - *Rationale:* The utility of multiple transluminal gateways to provide better drainage for patients with large necrotic collections treated using plastic stents has been previously established. Given that LAMS have wider lumen, there is likely no benefit to creating multiple tracts in patients with unilocular collections. However, in patients with DPDS, given the predisposition to prolonged illness and poor response to standard treatment, for collections >10 cm in size, a 15 or 20mm diameter LAMS and plastic stents were placed in two different transmural tracts. The objectives of this approach were to (a) establish better drainage (b) utilize the easily accessible LAMS tract for potential necrosectomy and (c) at follow-up, only the LAMS was removed and the plastic stents were left to indwell in order to drain the disconnected gland. The presence of indwelling plastic stents guarantee access to the necrotic cavity that can sometimes ‘seal off’ and preclude successful exchange of LAMS for plastic stents at follow-up. In patients with underlying DPDS and necrotic collections measuring less than 10cm in size, the relatively small size of the collection precludes multi-gate drainage. In these patients, the LAMS is exchanged for plastic stents at 3 to 4-week follow-up.   Although large bore drainage catheters can successfully drain necrotic collections, there is a significant risk for external fistula formation. Also, if the cavity is comprised predominantly of liquid contents, it may collapse completely following percutaneous drain placement. Therefore, our preference is always to establish internal drainage by endoscopic techniques and the external drain remains capped until internal transluminal endoprosthesis placement is accomplished.  **Drainage catheter management**   - *Plastic stent approach:* A nasocystic catheter was placed in patients with necrotic collections for irrigation and lavage. 14Fr percutaneous catheters were placed to drain collections that were endoscopically inaccessible. - *Integrated LAMS approach:* Nasocystic catheters were not used in any patient. 14Fr percutaneous catheters were placed to drain collections that were endoscopically inaccessible. - *Rationale:* Given that the wide lumen of LAMS facilitates better drainage and because the cavities were copiously irrigated and lavaged at index intervention, nasocystic catheters were not placed in the integrated LAMS cohort. Also, this approach minimizes patient inconvenience and reduces the need for frequent reinterventions due to dysfunctional or occluded nasocystic catheters.   **Follow-up protocol**   - *Plastic stent approach:* After discharge following the index intervention, a follow-up was scheduled at 8 weeks to assess treatment response. The endoprostheses were left to indwell in patients with DPDS and removed in patients with an intact main pancreatic duct. - *Integrated LAMS approach:* LAMS was removed (irrespective of the presence of DPDS) or exchanged for double pigtail plastic stents within a 4-week time frame. - *Rationale:* Given the increased risk for bleeding and buried stent syndrome in patients with indwelling LAMS, the stents were removed within a 4-week period. |
| --- |

**Table S2. Summary of treatment outcomes for necrotic collections only**

|  |  | **Plastic** | **Integrated LAMS** | **p-value** |
| --- | --- | --- | --- | --- |
|  |  | **(n=83)** | **(n=153)** |  |
| **Technical success: n (%)** | | 82 (98.8) | 153 (100) | 0.352 |
| **Treatment success: n (%)** | | 70 (84.3) | 144 (94.1) | 0.014 |
| **Recurrence: n (%)** | | 4 (4.8) | 6 (3.9) | 0.744 |
| **Reinterventions encompassing necrosectomy/additional endoscopic drainage: n (%)** | | 29 (34.9) | 55 (35.9) | 0.877 |
| **Rescue surgery: n (%)** | | 8 (9.6) | 3 (2.0) | 0.018 |
| **Adverse events: n (%)** | | 23 (27.7) | 39 (25.5) | 0.711 |
| **Mortality: n (%)** | All-cause mortality | 10 (12.0) | 12 (7.8) | 0.289 |
|  | From underlying disease/intervention | 6 (7.2) | 5 (3.3) | 0.201 |
| **Duration of hospital stay (days):** | Mean (SD) | 13.0 (22.8) | 10.5 (11.3) |  |
|  | Median | 4 | 6 | 0.234 |
|  | IQR | 2 - 14 | 2 - 15 |  |

**Table S3. Disease and procedure-related adverse events**

| **Type of disease-related adverse event** | **Plastic** | **Integrated LAMS** |
| --- | --- | --- |
| **Hematologic/hemorrhagic^a^: n (%)** | 8 | 16 |
| **Infection^b^: n (%)** | 16 | 12 |
| **Luminal^c^: n (%)** | 2 | 12 |
| **Neurological^d^: n (%)** | 0 | 1 |
| **Deaths: n (%)** | 8 | 6 |

a. Plastic cohort: Gastrointestinal bleeding (n=4) managed conservatively, DVT/PE (n=4).

Integrated LAMS cohort: Bleeding during insertion of cystogastrostomy stents (n=3 [LAMS =2, plastic =1]) managed conservatively, bleeding from LAMS cystogastrostomy site post-intervention (n=4) managed conservatively, bleeding during endoscopic necrosectomy (n=2) managed conservatively, bleeding from splenic artery pseudoaneurysm (n=3) managed with IR coil embolization in 2 and required coil insertion by vascular surgery in 1 patient, DVT/PE (n=4).

b. Plastic cohort: New onset SIRS/sepsis post-index intervention (n=11), pneumonia/pleural effusion (n=3), infection at PEG-J tube site/PEG-J tube malfunction (n=2).

Integrated LAMS cohort: New onset SIRS/sepsis post-index intervention (n=7), pneumonia/pleural effusion (n=4), infection at PEG-J tube site/PEG-J tube malfunction (n=1)

c. Plastic cohort: Perforation during placement of cystogastrostomy stents, which required surgical intervention (n=1), migration of cystogastrostomy stent, which required surgical intervention (n=1).

Integrated LAMS cohort: Migration of LAMS into PFC during stent deployment (n=2) requiring placement of second LAMS and retrieval of the migrated stent endoscopically in both patients, buried LAMS (n=2) requiring surgical removal in 1 and not removed in 1 patient, migration of LAMS out of PFC (n=3) requiring second LAMS placement to bridge the migrated stent in 2 and endoscopic removal in 1 patient, leakage of hydrogen peroxide into the abdominal cavity during irrigation phase of endoscopic necrosectomy (n=2) requiring abdominal washout, migration of PEG-J tube bumper into the abdominal cavity (n=2) resulting in gastric perforation in 1 patient and replaced endoscopically in 1 patient, enterocutaneous fistula (n=1) managed conservatively.

d. Integrated LAMS cohort: Cerebrovascular accident (n=1)
